# First opinion practice electronic health records are a useful source of descriptions of medication errors

**DOI:** 10.3389/fvets.2025.1560652

**Published:** 2025-05-20

**Authors:** Eirini Petrou, Heather Davies, Maria Aoun, Alan D. Radford, David Singleton, Peter-John M. Noble, David R. Killick

**Affiliations:** ^1^Department of Small Animal Clinical Science, Institute of Infection, Veterinary and Ecological Science, Leahurst, University of Liverpool, Liverpool, United Kingdom; ^2^Adams Vets, Liverpool, United Kingdom; ^3^Department of Population Medicine, Ontario Veterinary College, University of Guelph, Guelph, ON, Canada; ^4^Department of Infection and Microbiomes, Institute of Infection, Veterinary and Ecological Science, Leahurst, University of Liverpool, Liverpool, United Kingdom; ^5^IVC Evidensia, The Chocolate Factory, Bristol, United Kingdom

**Keywords:** adverse event, veterinary medicine, informatics, REGEX, general practice, patient safety, medical errors, pharmacovigilance

## Abstract

**Background:**

Medication error (MedE) is a leading global cause of harm in human healthcare with significance both in patient morbidity and mortality, and consequent legal and financial issues. Despite this, MedEs are a poorly explored area in veterinary medicine. Research has so far focussed on survey work and errors spontaneously reported to third parties, such as professional indemnity providers.

**Aim:**

Determine if MedEs can be successfully identified in first opinion electronic health records (EHRs).

**Animals:**

EHRs pertaining to animals treated in UK first opinion practice.

**Materials and methods:**

Regular expressions (REGEX) were designed (with assistance from a domain expert) to identify explicit reference to MedEs in the SAVSNET EHR dataset. Identified MedEs were then classified by the linear sequence of medication therapy, the degree of harm caused, the role of the person who made the error, and the medication type involved.

**Results:**

In total, 6,665 EHRs were identified by the REGEX, of which a random 2,847 were manually reviewed, with 1,023 (35.9%) matching the MedEs case definition. Of these MedEs, 29.5% (*n* = 302) caused mild harm to the patient, 2.8% (*n* = 27) moderate harm and 0.2% (*n* = 2) severe harm. MedEs were most frequent during the “drug administered” phase (51.4%) and within this phase, “dosing errors” were most common (68.1%). The most common medication types, associated with “drug administered” phase MedEs were vaccinations (27.1%) and non-steroidal anti-inflammatory drugs (19.0%).

**Conclusion:**

EHRs are a useful source of data on MedEs. MedEs are a common cause of patient harm in veterinary practice. The data provided here highlights drug classes at higher risk of problems for which mitigating action and/or education interventions are indicated.

## Introduction

In human medicine, medication errors (MedEs) are a leading cause of unnecessary harm globally (1). Beyond their direct effects on patients, they cause billions of dollars of financial losses (2), loss of patient trust, and significant emotional distress to clinical teams (3, 4) both directly due to the incidents and because of consequent litigation (5). Concerningly, the incidence of MedEs appears to be increasing, probably due to a combination of increasing life expectancy, drug innovation and associated poly pharmacy (6), and an increasing awareness of these issues leading to enhanced reporting. As might be expected, medical errors (including MedEs) are associated with feelings of guilt, loss of confidence and depression (7, 8).

MedEs are poorly explored in the veterinary context, but what little evidence there is suggests they may be common occurrences (4, 7) and account for up to 40% of all veterinary medical errors (9). The small number of previous studies which have investigated medical errors have used surveys (8), indemnity insurers records (2), poisons information centers (10) or voluntary online reporting systems (7, 9). Whilst electronic heath records (EHRs) may represent an additional and complementary source of data on MedEs, their use in veterinary medicine is yet to be explored in depth. Potential advantages may include more frequent recording of near-miss type events and events involving owners administering medications which may be poorly recorded in surveys or voluntary systems, due to a perceived or actual lack of harm. A further potential advantage of EHRs accruing data in real time is that issues peculiar to a novel product could be identified more promptly; such an approach using keyword searching of EHRs was shown to detect considerable numbers of MedEs in the human medical setting (11).

Previous studies have used various methods of classifying MedEs: (a) contextual classification which involves identifying time, place, medication, and persons involved; (b) modal classification which involves error types, such as overdose, wrong route, wrong drug etc. and (c) psychological classification which examines the root causes of why errors occurred and focuses on human sources of error (6). The nature of EHRs means that they are likely to be more suitable for identifying MedEs than investigating socio-psychological factors that increase risk of error; investigations of these factors is typically explored through qualitative approaches (e.g., surveys, interviews, and focus groups). Nonetheless, frequent errors highlight the potential need to enhance the safety features associated with a medication and EHRs may be well placed to provide such information.

We have previously shown that data regarding adverse drug reactions can be identified by rules-based searches of unstructured first opinion veterinary EHRs (29). In this paper we sought to expand our exploration of explicit recording of adverse drug events by (1) investigating the feasibility of identifying veterinary MedEs from unstructured first opinion veterinary EHRs (i.e., free-text, non-labelled clinical narratives) via a regular expression (REGEX)-based search, and (2) classifying identified MedEs by medicine group, type of error and severity.

## Methods

### UK first opinion electronic health records

EHRs pertaining to around 1.5 million pets from first opinion practices across the UK, collected between 1st March 2014 and 31st December 2020, were collated in the Small Animal Veterinary Surveillance Network (SAVSNET) database. SAVSNET recruits, by convenience, first opinion veterinary practices that are using compatible practice management software. The data is then collected in near real-time. The SAVSNET EHR dataset includes information about the animal (e.g., species, breed, sex, neuter status, age, owner’s postcode, insurance and microchipping status), as well as de-identified free text clinical narratives and prescribed treatments. SAVSNET data collection has been more completely described previously (12).

### Ethical approval

SAVSNET has ethical approval from the University of Liverpool Research Ethics Committee (RETH001081).

### Medication error case definition

Our case definition for a “medication error” was according to the National Coordinating Council for Medication Error Reporting and Prevention (NCCMERP) and was adapted from Ferner and Aronson (13), as follows: “any preventable event, recorded in the EHR clinical narratives reviewed, that may cause or lead to inappropriate medication use or patient harm while the medication is in the control of the health care professional, patient, or consumer. Such events may be related to professional practice, health care products, procedures, and systems, including prescribing, order communication, product labelling, packaging, and nomenclature, compounding, dispensing, distribution, administration, education, monitoring, and use”.

### Regular expression (REGEX) searches

REGEXs were developed to identify clinical narratives that might include a description of a MedE starting with the following word concepts: “mistake,” “error,” “wrong,” “incorrect” and “under/overdose.” A REGEX is a sequence of characters used to specify patterns to be matched within text. In addition to searching for specific character strings it allows for common variations (e.g., misspellings) or negations and facilitates searches by joining associated terms to together into a single large search term. An iterative approach was then taken to refine the REGEX, using associated words, word fragments and negation to develop a REGEX optimised for identifying these recorded MedEs. Each iteration was evaluated against 100,000 random clinical narratives. Those matching the REGEX (100 per REGEX) were reviewed by EP against the case definition and the positive predictive value (PPV) was calculated. The final REGEXs was chosen based on these PPVs.

### Medication error classification

**Figure 1:**
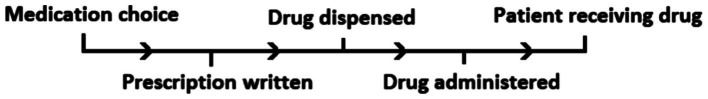
The “linear sequence of medication therapy” adapted from Barker et al. (14) and Aronson (6).

To classify the MedE types, an adapted version of a previously proposed “linear sequence of medication therapy” was used (6, 14) (Figure 1). This sequence represents a mixture of contextual and modal classifications, rather than a psychological classification. This decision was based on the nature of the data collected from EHRs which contain clinical events and are not specifically designed for psycho-social analysis of the root cause of MedEs, as such details are typically lacking in the clinical narrative.

The sequence of medication therapy begins from the point where a decision is made to prescribe a medication and continues through to the monitoring of the patient/assessment of response to therapy (see Figure 1). At any phase in the sequence, a MedE could occur.

Errors at each phase in the sequence were further subcategorised by the MedE type [adapted from Aronson (15)]:

“Medication choice” was classified into:

- Ineffective – medication prescribed is not indicated for the patient.
- Irrational and/or inappropriate – meaning the medication prescribed was chosen based on incomplete/missing information on the patient or irrational reasoning.
- Over/underprescribing – giving too high/low a dose of the medication for it to be effective, or not prescribing one which was indicated (6).

“Prescription written” was subcategorised into:

- wrong drug
- wrong dose
- wrong frequency

“Drug dispensed” was classified into:

- wrong drug
- wrong label
- wrong dose/amount

“Drug administered” into:

- wrong drug
- wrong dose/frequency/duration
- wrong route/application

“Patient receiving drug” (monitoring of the patient after the drug was prescribed) into:

- failure to monitor/alter
- failure to comply

Errors can therefore be considered in a manner analogous to directions. For example: “prescription written”/“wrong dose” reflects that when the drug was prescribed the prescribed the dose was incorrect. Whereas “drug dispensed”/”wrong dose” reflects that when the drug was dispensed the dispensed dose was incorrect. One classification was made per error. In a small number of cases more than one error was made in relation to one prescribed medication as detailed below.

Errors identified were further classified according to the degree of harm caused, adapted from the National Reporting and Learning System to complement veterinary clinical narratives (16); they were defined as seen in Table 1. A “near-miss” in the “No Harm” classification is defined as a medication which was intercepted before reaching the patient.

**Table 1:** Definitions of the “degree of harm” to a patient which could occur after a medication error is made.

Degree of harm	Definition
No harm	A medication error incident which resulted in no harm to the patient (including a near-miss);
Mild harm	A medication error incident which resulted in minor harm to the patient (e.g. vomiting and diarrhoea), required extra observation of the patient and/or a delay in treatment (including restarting a vaccination schedule);
Moderate harm	A medication error incident that resulted in: required hospitalisation of the patient caused significant but not permanent harm moderate increase in treatment cancelling of treatment or referral
Severe harm	A medication error that resulted in permanent harm or death

Adapted from the National Reporting and Learning System (15) to include a definition more relevant to veterinary practice.

Additionally, errors were classified based on: (a) who made the error (veterinary staff or the owner); (b) weight-related dosing errors (e.g., failure to monitor weight changes and alter dosing regimen) or (c) administrative errors (e.g., wrong history in medical notes etc.).

Finally, the medication types involved in the error (e.g., non-steroidal anti-inflammatory drugs, vaccines etc.) were also identified. A comparison was made between the proportion of errors for each medication group and the prescribed medications recorded within the entire SAVSNET dataset in the 2021 calendar year (a dataset fully annotated for drug sales was available for this year).

### Clinical narrative review

A random selection of the clinical narratives identified by the REGEX searches were first reviewed by EP and classified according to the system outlined above. As clinical narratives may include ambiguous descriptions, a subset of those narratives classified by the first reviewer were further reviewed by a second author (MA or DK) to determine the inter-reviewer variability in category assignment.

### Statistical analysis

Statistical analyses were completed using Microsoft Excel (Version 2011) and IBM SPSS Statistics 26. The incidence of identified MedEs was estimated as: (the proportion of reviewed REGEX hits containing a confirmed MedE * total number of REGEX hits) / total number of clinical narratives in the database during the period under study. The Kruskal-Wallis test was utilised to examine whether there was a difference between the categories of MedE types (independent variable with independent groups) and their frequency (dependent variable). Additionally, a Chi-Square test was used to examine whether there was a difference between MedE types and the frequency at which each type caused harm to a patient. Since the minimum number of observations is five, the degree of harm classifications were grouped together so that it was either “No harm,” or “Harm” caused to a patient. If statistical significance was found (*p* < 0.05), then post-hoc tests were completed using the Bonferroni correction to adjust for multiple comparisons.

## Results

### Assessment of PPV of REGEX designed to identify medication errors

A selection of base words were identified from which the REGEXs were developed. As it was considered likely that there would be a high number of irrelevant search hits, the final words and REGEX terms were selected based on PPV. The final REGEX for each keyword can be found in the Supplementary Data Sheet S1.

### REGEX based searches for the identification of MedEs

In total, 6,665 records were identified using the REGEX. A random 2,847 (42.7%) were manually reviewed and 1,023 (35.9%) met the case definition for a MedE and were subsequently classified. Common false positive hits were associated with missed negating terms (e.g., not overdosed), financial transactions or communication issues. In total, 1037 MedEs were identified (14 clinical narratives included two MedEs).

### Reviewer concordance

Secondary review found an overall reviewer concordance rate of 91% (*n* = 220/242) and similar concordance between clinical narratives categorised as containing an error (92%, *n* = 182/199) and those not containing one (88%, *n* = 37/42).

### Medication error incidence

In this SAVSNET dataset, the estimated incidence of MedEs identified by this rules-based approach was 3.5 per 10,000 clinical narratives.

### Veterinary staff were most frequently associated with MedEs

Of those 1,023 clinical narratives manually classified, veterinary staff made the error in 569 cases (55.6%) and owners in 340 cases (33.2%) (Table 2). No individual was identified as making the error in 118 cases (11.5%).

**Table 2:** The medication error type frequencies according to individual associated with the error for 1,037 MedEs.

	Veterinary staff	Owner	Not specified	Total
Medication choice	188	12	14	214
Prescription	85	0	3	88
Drug dispensed	97	0	30	127
Drug administered	170	302	56	528
Patient receiving drug	35	21	7	63
Unclear	4	5	8	17

### MedEs were most frequent during the drug administration step

There was an association between MedE types and their frequency (*p* < 0.001). “Drug administered” errors were most frequent, occurring in 50.9% of cases (*n* = 528), “medication choice” errors were next most common (*n* = 214, 20.6%), followed by “drug dispensed” errors (*n* = 127, 12.2%). The frequency of different subcategories of MedEs is shown in Table 3.

**Table 3:** Medication error category occurrence according to the “linear sequence of medication therapy” with percentage of each sub-category.

Category	*N*=	Sub-category	Percentage of category
Medication choice	214	Ineffective	11.7%
		Irrational/inappropriate	59.8%
		Over/under prescribing	28.5%
Prescription written	88	Wrong dose	58.0%
		Wrong drug	11.4%
		Wrong frequency	30.7%
Drug dispensed	127	Wrong drug	18.9%
		Wrong label	41.7%
		Wrong amount/dose	39.4%
Drug administered	528	Wrong drug	27.3%
		Wrong dose/frequency/duration	68.0%
		Wrong route/application	4.7%
Patient received drug	63	Failure to monitor/alter	41.3%
		Failure to comply	58.7%
Unclear	17	Unclear	100.0%

Of the 528 “Drug administered” errors, “wrong dose, frequency or duration of administration” was the most common classification accounting for 68.0% (*n* = 359) of this category. “Medication choice” errors were next most frequent (*n* = 214, 20.6%); with “irrational and/or inappropriate medication choice” (*n* = 128, 59.8%) being the most frequent subcategory. The vast majority of these were administration of an inappropriate vaccine (e.g., not following the planned protocol or administration of a vaccine intended for a different species). “Drug dispensed” errors were the third most common (*n* = 127, 12.2%), with the “wrong label” and “wrong dose/amount” being the majority. Of the “wrong dose/amount” errors, 34 (3.3% of total) related to patient weighing errors (which includes scale errors, failure to monitor weight changes, etc.).

### Medication type frequency varies with MedEs

The drug classes for which MedEs were most frequently identified were vaccination (*n* = 277, 27.1%), followed by nonsteroidal anti-inflammatory drugs (NSAIDs; *n* = 194, 19.0%), ectoparasiticides (*n* = 103, 10.1%), and antibiotics (*n* = 87, 8.5%). A summary of the number of MedEs by drug class is shown in Table 4.

**Table 4:** The frequency of medication types associated with medication error cases, in order of most to least frequent.

Drug class	*n*=	%
Vaccines	277	27.1%
NSAIDs	194	19.0%
Ectoparasiticides	103	10.1%
Antibiotics	87	8.5%
Wormers	49	4.8%
Thyroid	46	4.5%
Not specified	42	4.1%
Pain relief/premed/anaesthesia	31	3.0%
Cardiovascular	30	2.9%
Hormonal	29	2.8%
Atopy	26	2.5%
Anti-seizure	19	1.9%
Aural/topical	19	1.9%
Steroids	17	1.7%
GI/hepatic	12	1.2%
Other	10	1.0%
Opthalmic	8	0.8%
Urinary	7	0.7%
Anti-emetic	7	0.7%
Allergy	4	0.4%
Behavioral	4	0.4%
Supplements	2	0.2%

This has not been corrected for the relative use of each drug in practice compared to the rest; NSAIDs – non-steroidal anti-inflammatory drugs.

**Figure 2:**
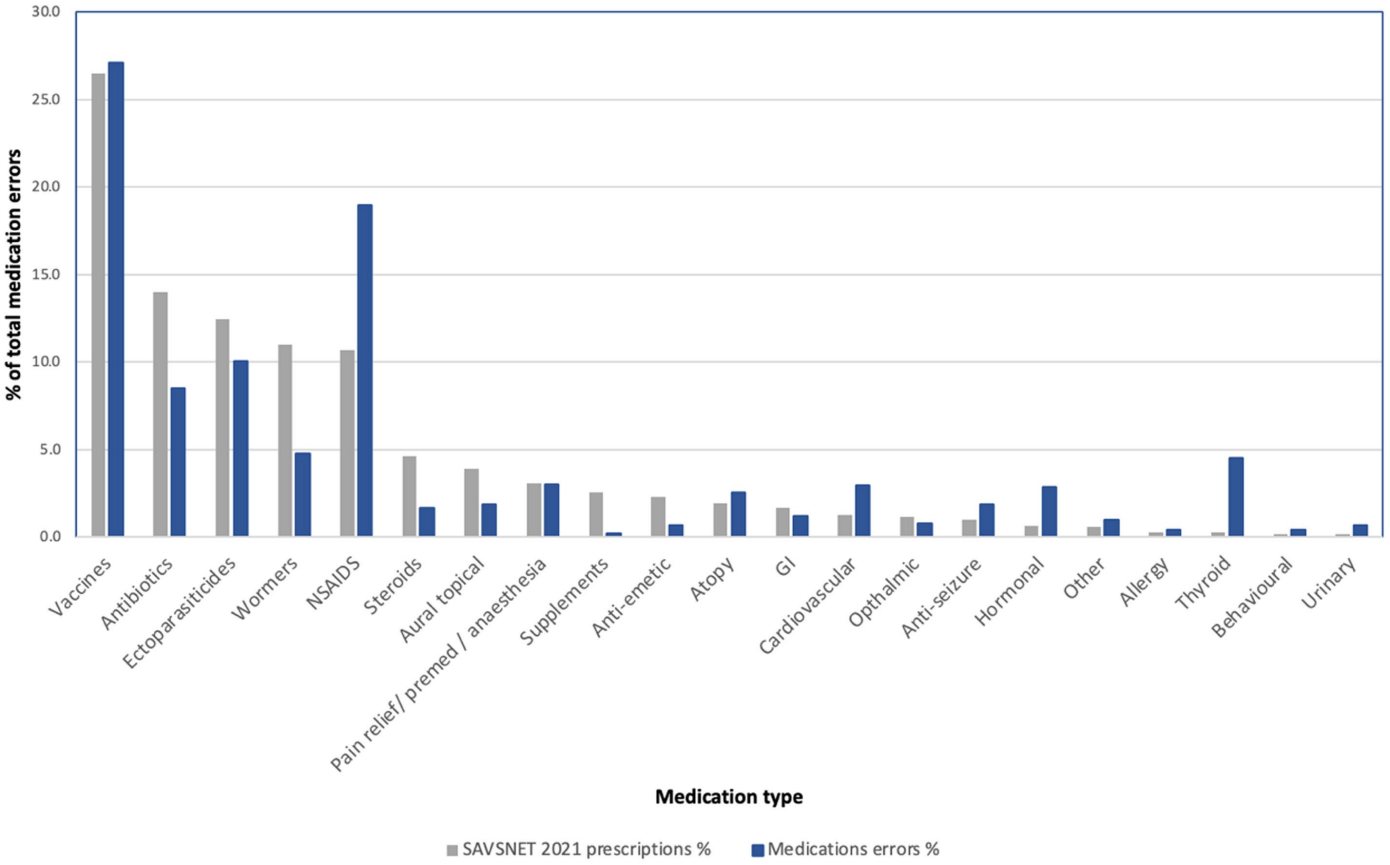
The percentage of medications by type in 1. prescribed medications in 2021 SAVSNET dataset (grey), 2. medication errors dataset (blue). NSAIDs – Non-steroidal anti-inflammatory drugs.

When the frequency of MedEs was compared to the frequency of prescription of each drug class in SAVSNET during the 2021 calendar year, it was noted that NSAIDs, hormonal medications, and urinary medication were relatively more frequent in the MedEs dataset versus the prescribed dataset (see Figure 2).

Of note medications dosed via owner prepared dosing syringe were identified frequently. In the NSAID class, 151 cases pertained to meloxicam, in the hormones group 15 pertained to insulin, and in the urinary group 4 of 7 MedEs were dosed via owner prepared dosing syringe.

### MedEs cause harm in veterinary practice

Of all 1,023 clinical narratives containing MedEs it was possible to determine the degree of harm in 1015 cases. In 67.4% (*n* = 684) cases there was no harm to the patient; of these 13.3% (*n* = 91) were a near-miss (a medication error intercepted before reaching the patient). Mild harm accounted for 29.7% of the errors (*n* = 301). Moderate harm was described in 28 cases in total (2.8%) and severe harm was caused in two cases (0.2%).

**Figure 3:**
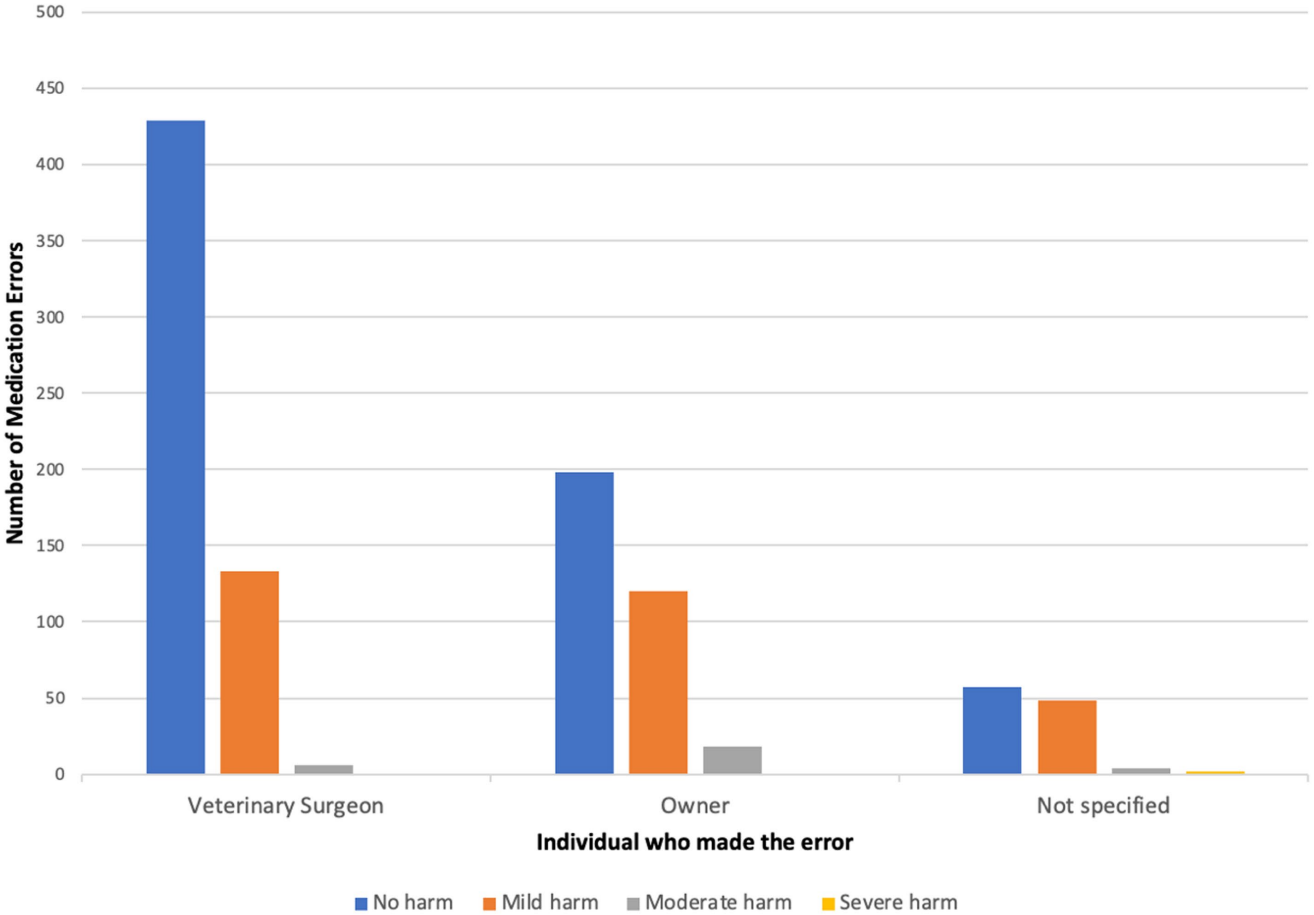
Shows the number of medication errors in each harm category broken down by individuals associated with the error.

The MedE type most frequently associated with moderate harm were “drug administration errors” (wrong dose/frequency/duration, *n* = 25). Of the 28 cases of moderate harm to the patient, 18 were associated with the owner, six related to veterinary staff and four cases did not specify any individual (Figure 3). The most mentioned drugs were NSAIDs (*n* = 18), and other pain relief/pre-medication (*n* = 5). Both MedEs associated with severe harm were due to “drug administration” errors (wrong dose/frequency/duration) and neither clinical narratives identified an associated person.

There was an association between MedE type and degree of harm (*p* < 0.001). MedE types relating to the “medication choice” and “drug dispensed” phases were associated with proportionally less harmful MedEs compared to MedEs in the “drug administered” phase (*p* < 0.001). MedEs during drug administration were more likely to be associated with harm compared to any other type (*p* < 0.001).

## Discussion

Here we show that searching clinical narratives at scale can efficiently identify consultations in which MedEs may have occurred. Manual annotation of retrieved records can then be used for classification of MedEs using contextual (error type, and the degree of harm) and modal methods (person who made the error, and the medication type associated). This paper describes over 1,000 recorded MedEs highlighting the most common types of error, and provides valuable data in a poorly explored, but important area of clinical practice.

It has been previously reported that MedEs are most frequently reported medical errors in veterinary practice and accounted for 40–69% of all medical errors (7, 9). These may be underestimates as cases which did not lead to harm may be less likely to be reported to voluntary reporting systems (11). A potentially useful feature of these data is therefore that near-misses and no harm errors are recorded in clinical narratives.

In this study we used a previously proposed “linear sequence of medication therapy” as a basis for describing the context in which the recorded MedEs occurred. We further used the data to explore which cohorts of individuals and groups of drugs were more commonly associated with particular error types and the harm caused by errors.

Drug administered phase errors were the most common group and of these, MedEs in which the *wrong dose* was administered were most frequent, accounting for 68.0% of all identified drug administration errors, which is a little higher than the figure of 54.7% found in a recent study (9). The frequency of errors due to the *wrong drug* was similar to two previous studies (2, 9). In a human study which also used EHRs, 65.2% of MedEs occurred during the drug administration phase and were due to the wrong dose/frequency/duration and 33.9% were due to the wrong drug (11). Similarly, two other studies reported that the most common error of drug administration was dose related, followed by the wrong drug (1, 17).

Unsurprisingly, the frequencies of the individual making the error varied by error type. The most common drug administration error for veterinary staff was the wrong drug which could mainly be accounted for by errors in vaccine selection. Errors associated with owners were most due to the wrong dose/frequency/duration. Identifying which errors are associated with particular groups allows for development of more targeted safeguards. For example owner errors might be reduced through optimised communication between veterinarians and their clients (2); through clear written instructions and/or drug preparation demonstrations for owners.

In this study, MedEs caused harm in 32.6% of cases (29.4% were mild harm, and approximately 3% were moderate or severe). This is similar to a recent veterinary study using a voluntary reporting system in which 28% of MedEs were associated with harm (27% harm and <1% death) (9). Studies in human healthcare have suggested somewhat similar results, with harm being associated with 20% and 33.2% of cases (6, 18). A further study in human health that used EHRs found that 41.3% of identified MedEs had an adverse outcome (11). Given that most Med Es are in the “drug administration” phase (i.e., relating to dosing amount and/or duration) the low level of harm likely reflects the relatively broad therapeutic window of most products with a license (which are likely to have the highest frequency of use) established during pre-marketing safety evaluations.

As noted above near misses may be less likely to be reported in a spontaneous reporting system and it is therefore noteworthy that around 13% of “No Harm” errors were classified as a near-miss, meaning the error could have caused significant harm had it not been intercepted (19). Identifying near misses may allow for mitigating action to be taken thus reducing MedEs in the future (20).

Apart from the harm to the patient, MedEs have a considerable financial and emotional impact for all those involved (4), including the potential to damage the relationship of trust between veterinarian and client. Human studies have taken steps to reduce these errors using various systems which have been demonstrated to reduce the frequency and impact on patients (20). Developing a safe environment requires understanding of the risk factors for errors. These include issues inherent to the medication, or the patient (especially in patients receiving multiple medications), and risks associated with staff and their working environment. In medical studies, various systems have been demonstrated to significantly decrease the frequency of unnecessary harm to patients. For example, the use of computerized physician order entry systems and clinical decision support tools for improving drug selection, resulted in a 70% reduction in adverse drug events (20). Other measures with a mitigating effect include medication reconciliation across more than one dispenser, a dedicated clinical pharmacist (21) (likely only feasible in large hospitals), bar-coding systems, and avoiding abbreviations (1). Automated warning systems are in development in human medicine and could provide dosing support during prescription calculations. Additionally, efforts made to increase clarity of medication branding or improve dosing instructions or devices would help users especially minimally trained (or untrained) owner users.

A novel feature of this work is that data on errors made by owners is available. This provides an additional facet for consideration of the context in which errors can occur. We found that errors involving preparing drug doses were more commonly associated with owners, however; as there is no prior information about owner involvement in MedEs it is impossible to draw a conclusion about how representative this data might be. Given the lack of clinical expertise of most owners it is hoped that they will seek appropriate guidance should an error occur, although this could be by contacting the manufacturer rather than the veterinary surgeon. It is however worth noting that concern about this risk has been raised by the FDA (22). Work in the human field has demonstrated that parents are able to prepare doses more accurately with dosing syringes than dosing spoons (23). Since preparing small doses of drugs in syringes remains a source of errors for clinically trained individuals (24) it seems logical to presume that performing this task in an accurate and repeatable way is also likely to be a challenge for pet owners. Preparing doses by syringe therefore remains a fallible improvement on other methods and more work is needed to determine if further improvements can be made to limit potential for error. In this context, a further stakeholder in MedEs are drug manufacturers which have a regulatory duty to conduct pharmacovigilance throughout the lifecycle of the product and a desire to maintain the best possible benefit: risk balance. Medication errors are a form of adverse drug event and frequent occurrence of particular types of MedEs for a given product may highlight a risk mitigation opportunity (e.g., reformulation of a product, increased educational efforts or automated reminders). Therefore, making drug manufacturers aware of common MedEs is an important element of professional pharmacovigilance activities. Moreover, this is particularly important for when products are being used (intentionally or otherwise) outside the label indication as drug manufacturers intrinsically have less safety data in this scenario.

A limitation of the data is that in order to be included, owner reports had to be both reported to a veterinary professional and then recorded in the EHR as a clinical narrative. This compares to a veterinary professional who can record MedEs directly. Within this study methodology we cannot determine if this process difference impacts the results. Moreover, it is feasible that veterinary professionals recording behavior may be influenced by circumstances. A related limitation is that the research data in SAVSNET is deidentified and so we could not further characterise the veterinary professional groups (i.e., veterinary surgeons, veterinary nurses etc.).

The purpose of this study was to evaluate whether MedEs can be identified in EHRs. The tool used for this was a rules-based (REGEX) search. The nature of rules-based searches is that there is an inherent interplay between precision and recall. Therefore, we focussed on five keywords that our initial evaluation suggested were likely to have a high PPV in our dataset. A consequential limitation of this study design is that it is very unlikely to capture all MedEs within the SAVSNET dataset. Additionally, certain keywords carry more information than just an error association. For example, under/overdose gave many cases of “drug administered” medication error types. Consequently, the selection of search terms in theory could bias the results regarding which phases in the linear sequence of medication therapy are more error prone. Moreover, ethical, societal and cultural factors such as shame, blame, a fear of liability or protection co-workers make it likely that not all errors are recorded (25, 26). Taking all these factors together it is clear that the incidence rate provided here can only be considered a minimum estimate and the true rate will be higher, however, as no prior work has provided an estimate of MedEs in first opinion practice we provide it here as starting point in this area of study.

A further consideration is that the REGEX will reflect the lexicon used within SAVSNET and consequently may need to be refined prior to use in studies in other datasets/domains. Nonetheless, we feel that these REGEXs provide researchers with a solid foundation should they wish to explore this area within their own datasets hence we provide them here. The increasing deployment of advanced informatics and artificial intelligence tools and the availability of large volumes of veterinary clinical data may allow for identification of a much larger cohort of MedEs which could not be identified using a rules-based approach (27, 28).

Inherent to the use of EHRs is that there are limited opportunities to clarify unclear or vague records. This means that there is an element of subjectivity in interpreting some of the clinical narratives, such as the degree of harm caused. Moreover, in some cases pertinent details were omitted (e.g., in relation to the two MedEs causing severe harm it was not possible to identify the associated individual). One route to reduce subjectivity would be an increased adherence to a standard nomenclature pertaining to different MedE types as has been developed for other clinical diagnosis (such as VENOM codes).

Due to data availability, we compared frequency of prescription in a single calendar year to the frequency of MedEs in our dataset (which relates to several years). This highlighted some drug classes associated with a greater risk of error. A limitation of this approach is that it requires an assumption that prescription rates for each class remained relatively constant throughout the study period. Finally, we note that SAVSNET recruits practices by convenience and so cannot necessarily be considered representative.

In conclusion, REGEX based searches of first opinion EHRs are an effective means of identifying clinical narratives which explicitly record MedEs, allow descriptive epidemiological analysis of their occurrence and are therefore a complimentary data source to previously described voluntary reporting systems. Whilst psycho-social root cause analysis is required to fully understand causative factors, details recorded in EHRs give a foundation on which interventions to mitigate against some future MedEs could be built. We identified particular classes of drugs at greater risk of particular errors, highlighting owner prepared dosing syringes being more frequently associated with error. The use of clinical narratives from EHRs therefore provides a foundation for identifying MedEs and testing interventions designed to mitigate and reduce them.

## Data Availability

The data analyzed in this study is subject to the following licenses/restrictions: the data for this article is derived from first opinion veterinary clinical narratives. For reasons of data protection (GDPR) and commercial interest of partners the primary data cannot therefore be released. Summary data can be made available on reasonable request. Requests to access these datasets should be directed to drk@liverpool.ac.uk.

## Supplementary material

SUPPLEMENTARY DATA SHEET S1 The regular expression (a sequence of characters that define a search pattern), created for each search word. For each regular expression, a positive predictive value (PPV) was calculated to assess its utility in identifying medication errors that matched the case definition and were included in the study accordingly. The test set PPVs were as follows: mistake (PPV=75%), under/overdose (65%), error (47%), wrong (44%), and incorrect (38%).
