# Supplementary material for: First opinion practice electronic health records are a useful source of descriptions of medication errors
Source: Front Vet Sci. 2025 May 20;12:1560652. doi: 10.3389/fvets.2025.1560652 (PMC12130827; doi:10.3389/fvets.2025.1560652)
Supplement: SUPPLEMENTARY DATA SHEET S1 — The regular expression (a sequence of characters that define a search pattern), created for each search word. For each regular expression, a positive predictive value (PPV) was calculated to assess its utility in identifying medication errors that matched the case definition and were included in the study accordingly. The test set PPVs were as follows: mistake (PPV=75%), under/overdose (65%), error (47%), wrong (44%), and incorrect (38%). [file Data_Sheet_1.PDF]

**EGEX**

```
ɔse|medication|\smeds?|prx|\Wrx|presc|scripts?|p[er]{1,2}sc?r?ip?tion|\svac{1,2}|bo{1,2}ster|worm(er|ing)).+)( (?<!no)(?<!not
\smistakes?)|((?<!no)(?<!not
\smistakes?)(.+(dose|medication|\smeds?\s|prx\W|rx\W|presc|scripts?\W|p[er]{1,2}sc?r?ip?tion|\svac{1,2}|bo{1,2}ster|worm(er|ing)))

ɔse|medication|\smeds?\s|prx|\Wrx|presc|scripts?|p[er]{1,2}sc?r?ip?tion|\svac{1,2}|bo{1,2}ster|worm(er|ing)).+)((?<!no)(?<!not an)(?<
?<!comput[e,o]r)(?<!price)(?<!pricing)(?<!booked in)(?<!machine)(?<!avoid)(?<!avoiding)(?<!scale)(?<!human)(?<!thermometer)(?<!could
\ser{1,2}ors?(?!\smessage))|((?<!no)(?<!not an)(?<!trial and)(?<!trial &)(?<!comput[e,o]r)(?<!price)(?<!pricing)(?<!booked
?<!machine)(?<!avoid)(?<!avoiding)(?<!scale)(?<!human)(?<!thermometer)(?<!could be
\ser{1,2}ors?(?!\smessage))(.+(dose|medication|\smeds?\s|prx\W|rx\W|presc|scripts?\W|p[er]{1,2}sc?r?ip?tion|\svac{1,2}|bo{1,2}ster|

ɔse|medication|\smeds?|prx|presc|scripts?|p[er]{1,2}sc?r?ip?tion|\svacc?|bo{1,2}ster|worm(er|ing)).+)((?<!thing)(?<!thing is)(?<!thing
s)(?<!really)(?<!obviously)(?<!anything else)(?<!what is)(?<!what's)(?<!what was)(?<!not)(?<!not be)(?<!very)(?<!could be)(?<!would
(?<!clinically)(?<!appears)(?<!or)(?<!charged)(?<!booked in)(?<!batch number)(?<!\sbn)(?<!no)(?<!things
ng)(?<!much)\swrong(ly)?(?!\s(position(ing)?|price|time|thing|places?|side|angle|chip|code|batch|bn\s|add?ress)))|((?<!thing)(?<!thin
s)(?<!really)(?<!obviously)(?<!anything else)(?<!what is)(?<!what's)(?<!what was)(?<!not)(?<!not be)(?<!very)(?<!could be)(?<!would
(?<!clinically)(?<!appears)(?<!or)(?<!charged)(?<!booked in)(?<!batch number)(?<!\sbn)(?<!no)(?<!things
ng)(?<!much)\swrong(ly)?(?!\s(position(ing)?|price|time|thing|places?|side|angle|chip|code|batch|bn\s|add?ress)))(.+(dose|medicatio
|scripts?\W|p[er]{1,2}sc?r?ip?tion|\svacc?|bo{1,2}ster|worm(er|ing)))

ɔse|medication|\smeds?\s|prx|\Wrx|presc|scripts?|p[er]{1,2}sc?r?ip?tion|\svac{1,2}|bo{1,2}ster|worm(er|ing)).+)((?<!electrolytes\W)(?
V)(?<!\Wbn\W)(?<!batch number\W)(?<!batch no\W)(?<!batch no\.\W)(?<!age may
,W)incorr?ect(ly)?(?!\W* [illegible] ɜs\\W)(?<!cha
\Wbn\W)(?<!batch num [illegible]
,W)incorr?ect(ly)?(?!\W* [illegible] ication|\smed
er]{1,2}sc?r?ip?ti?on|scr [illegible]

!could)(?<!may)(?<!risk of)(?<!risk)(?<!no)(?<!not to)(?<!not)(?<!want to)(?<!if)(?<!case of)(?<!case)(?<!easy to)(?<!difficult to)(?<!hard
(?<!don't)(?<!dont)(?<!do not)(?<!avoid)(?<!Pent[a,o]ject)(?<!Pent[a,o]ject IV)(?<!pentobarbit[a,o]l)(?<!pentobarb)\s(over|under)dose(d|i
ıt[a,o]ject)
```
